# Development and characterization of ivermectin nanoformulations for topical acaricidal activity against *Rhipicephalus sanguineus* ticks

**DOI:** 10.1038/s41598-026-56357-0

**Published:** 2026-06-12

**Authors:** Hoda S. M. Abdel-Ghany, Sobhy Abdel-Shafy, Amany M. Mohamed, Abeer S. Hassan

**Affiliations:** 1 Department of Parasitology and Animal Diseases, Veterinary Research Institute, National Research Centre, 33 Bohouth St., Dokki, Giza, 12622 Egypt; 2 Ticks and Tick-Borne Diseases Research Unit, Veterinary Research Institute, National Research Centre, Dokki, Giza, 12622 Egypt; 3 Department of Pharmaceutics, Faculty of Pharmacy, Qena University (South Valley University), Qena, 83523 Egypt; 4 Department of Pharmaceutics and Pharmaceutical Technology, Faculty of Pharmacy, Badr University in Assiut, Naser City, Assiut 2014101 Egypt

**Keywords:** Acaricide, Ivermectin, Ticks, Control, Nanoformulations, Spanlastics, Biological techniques, Diseases, Zoology

## Abstract

Ticks are among the most significant ectoparasites of livestock and humans, posing serious health and economic risks. The growing resistance to conventional acaricides highlights the need for safer and more effective alternatives. This study aimed to evaluate the acaricidal efficacy of different ivermectin (IVM) nanoformulations against different developmental stages of *Rhipicephalus sanguineus* tick. Different ivermectin nanoformulations were fabricated and evaluated for their physicochemical properties. An in vitro study was performed using larval, nymphal, and adult immersion tests, followed by an in vivo trial against unfed adults using the most effective formulation. All nanoformulations showed particle sizes from 450 to 650 nm and a polydispersity index range from 0.34 to 0.65. IVM spanlastics exhibited the smallest particle size, highest encapsulation efficiency (92%), and a sustained release profile. Based on LC_50_ values, IVM spanlastics showed superior acaricidal activity against larvae (LC_50_: 0.05%), nymphs (LC_50_: 0.09%), and unfed adults (LC_50_:0.16%); followed by IVM-SeNPs, where the LC_50_ values were 0.10, 0.23, and 0.38% for larvae, nymphs, and unfed adults, respectively. The in vivo study of IVM spanlastics demonstrated 100% mortality of adult ticks within four days after application. IVM spanlastics could be topically applied as an alternative to conventional injectable IVM to control *R. sanguineus* ticks. Further toxicological studies are necessary to ensure the safety of these formulations for environmental and veterinary use.

## Introduction

Ticks are obligate hematophagous ectoparasites that feed on the blood of livestock, pets, and humans^1,2^. Ticks cause anemia, a reduction in weight gain and milk, as well as injuries in the skin of animals^3,4^. Ticks play a crucial role in transmitting infectious diseases to animals^5^. They are vectors for various pathogens causing diseases such as babesiosis, theileriosis, anaplasmosis, borreliosis, rickettsiosis, and ehrlichiosis^6,7^. The brown dog tick, *Rhipicephalus sanguineus* sensu lato (Latreille, 1806) (Acari: Ixodidae), is a three-host tick that primarily parasitizes dogs and other carnivores worldwide^8^. It occasionally infests mammals and humans, highlighting its broad host range^9,10^.

Ticks are usually controlled by chemical acaricides, which are expensive, environmentally hazardous, and may result in residues in animal products^4^. Common acaricides typically contain neurotoxic compounds, such as organophosphates, organochlorines, and pyrethroids. Additionally, systemic parasiticides administered orally or topically to hosts are also used to control tick infestations^11–14^. Resistance of ticks to various synthetic pesticides has become widespread due to the overuse of chemical acaricides^15^. To mitigate this issue, current tick control programs recommend rotating acaricides to reduce the emergence of a resistant tick strain^16^.

Integrated vector management approaches, incorporating alternative measures with or without chemical insecticides, have shown promise in effectively controlling tick populations and tick-borne diseases^4,17^. Many studies have evaluated natural products, nanomaterials, and entomopathogenic nematodes as alternative strategies in tick control on/off hosts and have promising results as acaricidal efficacy^17–21^.

Synthetic acaricides remain the primary method for tick control and will likely continue to be the cornerstone of control programs in the coming years, emphasizing the need for strategic use^22^. Furthermore, nanocarriers can improve tissue uptake and distribution while reducing toxicity, making them an attractive delivery method for veterinary pest control^23–26^.

Ivermectin (IVM), which belongs to the avermectin category, is a crucial drug endorsed by the WHO since 1981^27^. IVM is classified as a Class II compound according to the Biopharmaceutical Classification System (BCS), indicating poor aqueous solubility of ~ 4 µg/mL at 25 °C and a high partition coefficient log P of 3.2^28^. Ivermectin is an antiparasitic drug and is mainly administered via injection. However, this conventional method often faces limitations related to dose toxicity and the inability to target free-living stages of ticks in the environment. By using nanotechnology, this study promotes a shift toward topical delivery methods, such as immersion baths and spraying, as alternatives to traditional injections. This approach allows for direct control of *R. sanguineus* during its various life stages and reduces dependence on systemic treatments. Accordingly, the formulation of ivermectin has been challenged because of its incomplete absorption, fluctuating, and low oral bioavailability. The researcher in previous reports investigated new tools to overcome the main shortcomings regarding limited solubility and low oral bioavailability. For example, the utilization of nanoparticulate based drug delivery systems (liposomes, niosomes, transferosomes, solid lipid nanoparticles, and nanostructured lipid carriers)^29^. On the other hand, topical applications of ivermectin in new formulations would be highly advantageous due to minimizing the adverse effects of oral ivermectin and achieving higher localization on the skin. Consequently, it delivers a strong therapeutic effect directly on the site of action^28,30^. For parasites located in self-assembly, non-ionic surfactant-based nano-formulations offer several benefits over conventional topical preparations, including enhanced drug solubility, improved drug accumulation in the skin, and sustained drug release, which may lead to reduced dosing and frequency of topical administration.

Therefore, this study aimed to fabricate different IVM nanoformulations using different nanocarriers: IVM spanlastics, IVM selenium nanoparticles (IVM Se-NPs), IVM chitosan nanoparticles (IVM Cs-NPs), and IVM selenium chitosan nanoconjugate (IVM Se-Cs-NCJ), to evaluate their acaricidal activity against different developmental stages of *R. sanguineus* tick compared with free IVM.

## Materials and methods

### Ethical statement

This study received approval from the Ethical Committee for Medical and Veterinary Research at the National Research Centre (NRC), Egypt (approval protocol No. 05410325). All procedures, including animal welfare and ethical considerations, were conducted in accordance with relevant guidelines and regulations. Furthermore, all methods were reported following ARRIVE guidelines.

### Chemicals

Ivermectin was obtained from UNIPHARMA, Co., Egypt. Span 60 was obtained from Sigma-Aldrich Co. (St. Louis, MO, USA), while Tween 40 and ethanol were obtained from El-Nasr Pharmaceutical Chemicals Co. (Cairo, Egypt). Chitosan (CS), low molecular weight (The degree of deacetylation is 98%), was purchased from Industrial Manufacturing Co., Tokyo, Japan. Sodium selenite (the salt which is used for the preparation of selenium nanoparticles) was purchased from Sigma Aldrich (St. Louis, MO, USA).

### Fabrication of ivermectin-loaded chitosan nanoparticles

Chitosan nanoparticle formulation loaded with ivermectin was prepared using the ion gelation method. The procedures were performed according to Iswanti et al.^31^. In brief, the chitosan solution was made by dissolving chitosan in 1% acetic acid to achieve a concentration of 0.4%. The pH was adjusted to 4.5 with 1 M NaOH. Next, 10 mL of the chitosan solution was placed on a magnetic stirrer at 3000 rpm at room temperature overnight. Ivermectin (0.5%), dissolved in PEG400, was mixed with 5 mL of 0.2% sodium tripolyphosphate (TPP) aqueous solution. The chitosan solution (10 mL) was slowly added dropwise into the TPP-ivermectin mixture with a syringe at a rate of 0.5 mL/min. The resulting colloidal dispersion of ivermectin-loaded chitosan nanoparticles was sonicated (Acculab Ultrasonic, USA) for 10 min with three seconds on/off cycles at 50% amplitude. The final formulation was stored in a refrigerator for further studies.

### Formulation of ivermectin-loaded spanlastics

Ivermectin nanovesicles were fabricated, displaying the ethanol injection technique as reported in previous literature^32,33^. Briefly, measured amounts of ivermectin and span 60 were dissolved in a small volume of ethanol and then slowly injected dropwise into a preheated aqueous solution of Tween 40 at 60 °C (Span 60: Tween 40 was used at a weight ratio of 2:1). The solution was continuously stirred on a magnetic stirrer (Thermo Scientific and SA Co., China) for 30 min at 600 rpm until all organic solvent evaporated and the IVM spanlastic dispersion formed. The prepared formulation was stored in a refrigerator (4 °C) overnight for further use.

### Green synthesis of selenium nanoparticles and loading of ivermectin

Selenium nanoparticles were fabricated using a green method according to a previous study^34^. A novel green chemistry synthesis technique was demonstrated for producing Se-NPs by reducing sodium selenite with an aqueous solution of quercetin. Briefly, the quercetin solution was prepared by dissolving a specific amount in 50 mL of distilled water. The aqueous quercetin solution was added dropwise at a fixed stirring rate to 50 mL of a 10 mM sodium selenite solution maintained at 50 °C. The mixture was stirred for 24 h on a magnetic stirrer to ensure complete reduction and formation of Se-NPs. The color changed from bright yellow to orange-red, indicating the production of Se-NPs. The resulting nanoparticle dispersion was then stored in a refrigerator for further investigation. The calculated amount of ivermectin solution was added to the selenium NPs dispersion to prepare IVM-loaded SeNPs at a concentration of 0.5%.

### Loading of ivermectin into chitosan-selenium NPs mixture to prepare IVM nanoconjugate

Chitosan solution was mixed with selenium nanoparticles at a ratio of 1:1 on a magnetic stirrer till homogenous dispersion was developed and stored for further studies. Ivermectin solution was added slowly into the mixture of chitosan-selenium NCJ to prepare the nanoconjugate of IVM at a concentration of 0.5% w/v.

### In vitro characterization of ivermectin nanoformulations

#### Determination of the drug content and entrapment efficiency

Methanol was utilized to dissolve the developed formulation. The total drug content of the produced dispersion was measured by dissolving 0.2 ml of the colloidal dispersion in 15 ml of methanol, followed by estimating the drug concentration using a UV-Visible spectrophotometer (LISCO GmbH Bargteheide, Germany) at the λ max of ivermectin (245 nm)^35^. Entrapment efficiency (EE%) was determined indirectly by centrifuging the nanodispersions at 18,000 rpm for 0.5 h (chitosan NPs) and 1 h (spanlastics vesicles) with a cooling centrifuge (Beckman, Fullerton, Canada) at 4 °C. A UV-Vis spectrophotometer was used to measure the concentration of free drug present in the collected supernatant. The EE% was calculated using the following equation^36^:

1 $$\:\%EE=\frac{Total\: drug\: amount-Free\: drug\: amount\:}{Total\: drug\: amount}x\:100$$

To calculate the drug loaded onto selenium nanoparticles, an indirect method was used to estimate the amount of ivermectin after centrifugation at 8000 rpm, and then the free drug was measured in the supernatant at λ max of 245. Equation (1) was applied to measure the entrapment efficiency.

The drug (IVM) that is conjugated to the mixture of chitosan-selenium NPs was calculated by the same aforementioned indirect method. All the determinations were made in triplicate and presented as the mean of three values ± SD.

#### Determination of vesicle size, polydispersity index, and zeta-potential

The average particle size, polydispersity index (PDI), and zeta potential of the fabricated ivermectin nanoformulations were determined using a Malvern Zetasizer Nano series ZS instrument (Malvern Instruments, Malvern, UK) after appropriate dilution at room temperature^37^. Measurements were conducted in triplicate, and results are presented as mean ± SD of three independent measurements.

#### Assessment of morphology

The morphology of the prepared IVM selected nanoformulation (nanospanlastics) was examined using a Transmission electron microscope (JEOL 100 CX II, Tokyo, Japan). A drop of diluted dispersion was placed on a carbon-coated copper grid and allowed to adhere for 1 min. After air-drying, the sample was photographed^38^.

#### In vitro drug release studies

The in vitro release profile of ivermectin from nanoformulations was evaluated using a dialysis method across a cellophane membrane (molecular weight cutoff 12,000–14,000, Sigma Aldrich, St. Louis, MO, USA), as previously described by Hassan and Soliman^39^. In brief, 1 ml of the tested formulation (0.5% w/v ivermectin) was weighed and placed on a previously soaked cellophane membrane fixed at the lower open end of a glass tube. An equal amount of free drug suspension was placed into another tube and used for comparison. The glass tubes were immersed in a beaker containing 100 mL of phosphate buffer (pH 6.8) maintained at 37 °C. The beakers were placed in a thermostatically controlled shaking water bath (DAIHAN Scientific Co., Seoul, South Korea) and agitated at 50 rpm. At predetermined intervals (0.5, 1, 2, 4, 6, 12, 24, and 48 h), 5 mL samples were withdrawn and replaced with an equal volume of fresh medium. Drug content was quantified using a UV-Visible spectrophotometer at λ max 245 nm. All measurements were performed in triplicate.

### Release kinetic analysis

The mechanism of drug release was evaluated by fitting data to various kinetic models: zero-order, first-order, Higuchi diffusion, and Korsmeyer–Peppas equation^38^.

#### Zero-order kinetics

$${\mathrm{Q}} = {\mathrm{k}}_{0} {\mathrm{t}}$$

Where **Q** is the drug released at time t^40^.

K_0_ is the zero-order release constant.

t: refer to time.

#### First-order kinetics

$$\ln \left( {100 - {\mathrm{Q}}} \right) = \ln 100 - {\mathrm{k}}_{1} {\mathrm{t}}$$

Where K_1_ is the first-order release constant^40^.

#### Higuchi model

$${\mathrm{Q}} = {\mathrm{k}}_{{\mathrm{H}}} {\mathrm{t}}^{{1/2}}$$

Where Q is the amount of drug released at time t per unit area^41^.

KH is the Higuchi release rate constant.

#### Korsmeyer-Peppas equation

$${\mathrm{Mt}}/{\mathrm{M}}\infty = {\mathrm{ktn}}$$

Where Mt/M ∞ is the fraction of drug released at time t......

n is the release exponent.

### Ticks

The different developmental stages of *R. sanguineus* ticks were obtained from an established colony reared on rabbits at the Parasitology and Animal Diseases Department, Veterinary Research Institute, National Research Centre, Dokki, Giza. Engorged females were incubated at 25 ± 1 °C and 75–80% relative humidity (RH) till the end of oviposition. After hatching, a portion of the larvae was used in the bioassay, and the other portion was fed on healthy rabbits to obtain fully engorged larvae, which were incubated for molting to unfed nymphs. A portion of unfed nymphs was used in the bioassay, and other portions were fed on rabbits to obtain fully engorged nymphs, which were incubated to obtain unfed adults that were used in the bioassay according to Abdel-Ghany et al.^18^.

### In vitro evaluation of ivermectin nanoformulations

The IVM nanoformulations (IVM spanlastics, IVM Se-NPs, IVM Cs-NPs, and IVM Se-Cs-NCJ, free IVM) were diluted in distilled water to obtain five different concentrations of 0.5, 0.25, 0.125, 0.0625, and 0.0313%. These concentrations were chosen based on a pilot study. The control group was treated with distilled water and a nanocarrier without IVM.

Free ivermectin was prepared to compare its efficacy with nanoformulations that contain highly solubilized form of ivermectin. In brief, the weighed amount of ivermectin was added to the aqueous media under magnetic stirring at high speed (3000 rpm) till the drug dispersion was obtained. All the preparations were composed of the same starting amount of ivermectin.

### Larval immersion test (LIT)

The LIT was performed according to Abdel-Ghany et al.^19^ with slight modifications. A micro-centrifuge tube was used to immerse approximately 100 larvae in 200 µl of the tested materials for 2 min. Three replicates were used for each concentration (100 larvae per replicate). After immersion, the larvae were transferred using a fine brush to plastic tubes containing filter paper covered with muslin gauze, and they were examined after 24 h to record mortality. The larvae were examined by naked eye and under a stereomicroscope, and they were considered dead when gently stimulated with a fine brush and were unable to move.

### Nymphal immersion test (NIT)

Unfed nymphs were immersed in a microcentrifuge tube containing 1 ml of the test materials for 2 min and then dried on filter paper. Next, they were placed in plastic cups covered with muslin gauze and observed for 3 consecutive days. Thirty unfed nymphs were used for each concentration and divided into 3 replicates (10/replicate). Nymphs were examined by naked eye and under a stereomicroscope and considered dead when they were stimulated with a fine brush and unable to move.

### Adult immersion test (AIT)

Thirty unfed adults were allocated into 3 replicates (*n* = 10/replicate) and immersed in 5 ml of the tested concentrations for 2 min according to Abdel-Ghany et al.^42^. After immersion, they were dried on filter paper, transferred into plastic cups, incubated, and examined over 3 successive days. The mortality of unfed adults was assessed by naked eye and under a stereomicroscope. Ticks that showed no movement when gently stimulated with a fine brush and failed to respond were considered dead.

### In vivo evaluation of ivermectin spanlastics on rabbits

Based on the in vitro study, the most effective material (IVM Spanlastics) against all developmental stages of ticks was selected for the in vivo study. For this experiment, six - 4–6-month-old healthy male New Zealand white rabbits (average weight: 2–2.5 kg) were housed at the National Research Centre animal house. Initially, all rabbits were experimentally infested with an equal number of unfed male and female *R. sanguineus* ticks (20 males and 20 females) using the capsule technique. Afterward, the rabbits were divided into two groups (3 rabbits per group). Group (A) received ivermectin spanlastics treatment, while Group (B) was treated with distilled water. Two days after tick infestation, Group (A) was sprayed with 0.5% ivermectin-loaded spanlastics, and Group (B) was sprayed with distilled water. Each rabbit was sprayed only once with 2 ml of the tested material or distilled water. The acaricidal efficacy of the tested spray was monitored at 24, 48, 72, and 96 h after exposure.

### Statistical analysis

The mortality percentages of larvae, nymphs, and unfed adults treated with tested materials were compared using one-way ANOVA and Tukey’s HSD tests (SPSS version 20). LC₅₀ values were calculated via regression analysis of probit-transformed mortality data. Dose-response relationships were analyzed by the probit method^43^ using Ldp lineR software (Ehabsoft) to assess concentration-dependent effects.

## Results

### Preparation and characterization of different ivermectin nanocarriers

Different nanocarriers of IVM (IVM spanlastics, IVM Se-NPs, IVM Cs-NPs, and IVM Se-Cs-NCJ) were successfully prepared and characterized, with the results illustrated in Table 1. As observed, all the formulations showed particle sizes in the nanometer range from 450 to 650 nm according to the results of dynamic light scattering. It was observed that IVM spanlastics showed significantly (*p* ≤ 0.05) smaller particle sizes (450 nm) and lower PDI values (0.34) compared to other nanoformulations. Furthermore, the measured zeta potential of the fabricated nanocarriers ranged from − 8.5 to -30 mV, with the highest value obtained with the spanlastics formulation. The encapsulation efficiency of the developed ivermectin spanlastic was found to be 92 ± 1.8%, while other nanoformulations displayed significantly lower values of conjugation and encapsulation efficiency compared to vesicular spanlastics (Table 1).

**Table 1:** Physicochemical characterization of different ivermectin-loaded nanocarriers. Data are presented as mean ±SD (n=3)

Formulation	Particle size (nm)	Zetapotential (mV)	PDI	Encapsulation or conjugation efficiency %
IVM spanlastics	450 ± 11.5	-30 ± 1.9	0.34 ± 0.01	92 ± 1.8
IVM selenium NPs	600 ± 15.2	-8.5 ± 0.6	0.45 ± 0.06	38 ± 1.6
IVM chitosan NPs	650 ± 10.6	-10 ± 1.2	0.65 ± 0.02	40 ± 2.4
IVM selenium-chitosan NPs	860 ± 19.1	-15 ± 2.6	0.55 ± 0.02	35 ± 2.1

**Fig. 1:**
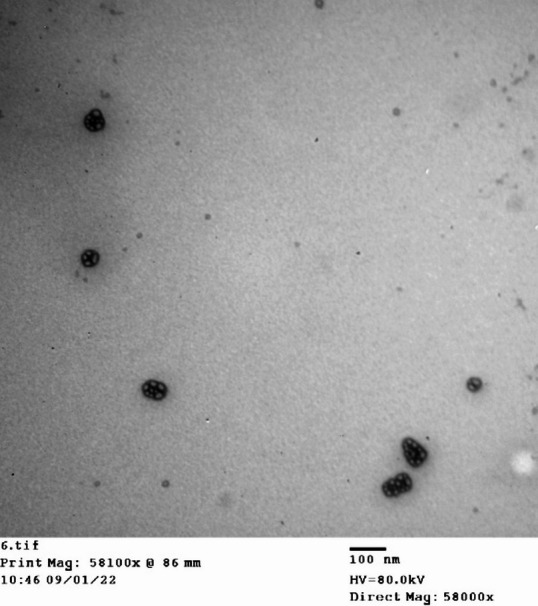
TEM image of the selected formulation ivermectin spanlastics.

**Fig. 2:**
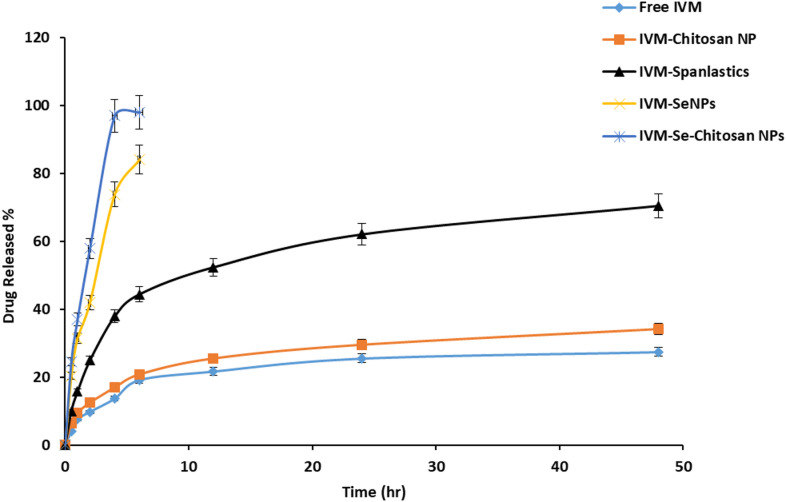
Cumulative in-vitro release profiles of ivermectin from different nanocarriers and free drug solution in phosphate buffer pH 6.8 at 37 °C. Data are presented as mean ± SD (*n* = 3).

Figure 1 shows the morphological image of the selected formulation (IVM spanlastics). As observed, the nanocarriers are in the nanosized range, spherical in shape, and homogeneously distributed. IVM release from the prepared formulations was evaluated using a dialysis diffusion method across a cellophane membrane, in phosphate buffer (pH 6.8, 37 °C) over 48 h. The release profiles are depicted in Fig. 2. The developed nanocarrier formulations exhibited an initial burst release of IVM from spanlastics and Cs-NPs, followed by sustained release over 48 h. Notably, IVM Se NPs and IVM Se-Cs-NCJ showed a more rapid initial release and significantly higher (*P* < 0.05) drug release rates 98% and 84% after 48 h from Se-Cs and Se-NPs, respectively, compared to spanlastics, Cs-NPs, and free IVM suspension. As observed, the developed formulations demonstrated better release profiles compared to free IVM suspension (only 27.4% after 48 h) (Fig. 2). Ivermectin release data were fitted to various kinetic models (Zero-Order, First-Order, Higuchi-Diffusion, and Korsmeyer-Peppas), with results summarized in Table 2. The model best describing the release mechanism was selected based on the highest correlation coefficient (R²). The examined formulations showed the highest R² values for the Higuchi diffusion model, indicating diffusion-controlled release.

**Table 2:** Release kinetic results of the investigated ivermectin nanocarriers.

Formulation	Zero-order model (*R*^2^)	First order model(*R*^2^)	Higuchi diffusion model (*R*^2^)	Korsmeyer-Peppas model (*R*^2^)	Korsmeyer-Peppas model (*n*)
IVM spanlastics	0.845	0.917	0.944	0.986	0.541
IVM selenium NPs	0.844	0.889	0.962	0.993	0.520
IVM chitosan NPs	0.873	0.895	0.948	0.985	0.367
IVM selenium-chitosan NPs	0.785	0.844	0.888	0.999	0.60

### In vitro efficacy of ivermectin nanoformulations

Four nanoformulations of IVM, along with free IVM, were in vitro evaluated against larvae, nymphs, and unfed adults of *R. sanguineus* ticks, compared with the control. Overall, mortality rates of all tick stages increased with increasing concentrations and time across all tested formulations. The lowest concentration (0.0313%) in all formulations did not cause any mortality in all tick stages. In contrast, the remaining four concentrations (0.5, 0.25, 0.125, and 0.0625%) resulted in varying degrees of mortality depending on the tick stage and exposure duration (Tables 3, 4 and 5).

**Table 3:** Mortality percentages (Mean ± SE) of *Rhipicephalus sanguineus* larvae 24 h post exposure to ivermectin and its different nano-formulations.

Formulation	Concentrations (%)			
	0.5	0.25	0.125	0.0625
IVM spanlastics	100 ± 0.0^a^	88.7 ± 4.7^a^	82.8 ± 2.8^a^	52.5 ± 6.3^a^
IVM selenium NPs	95.9 ± 4.1^a^	73.3 ± 1.9^b^	56.2 ± 3.2^b^	33.9 ± 2.8^b^
IVM chitosan NPs	86.3 ± 1.0^ab^	56.7 ± 1.6^c^	35.6 ± 6.1^c^	18.5 ± 2.9b^c^
IVM selenium-chitosan NPs	71.5 ± 4.7^bc^	58.2 ± 2.2^c^	53.0 ± 1.6^bc^	25.5 ± 4.0^bc^
Free IVM	56.7 ± 6.7^c^	54.5 ± 2.3^c^	42.0 ± 5.3^bc^	32.4 ± 1.1^b^
Nano-carrier without IVM	8.3 ± 1.7^d^	8.3 ± 1.7^d^	8.3 ± 1.7^d^	8.3 ± 1.7^c^
Control (D.W)	8.4 ± 4.3^d^	8.4 ± 4.3^d^	8.4 ± 4.3^d^	8.4 ± 4.3^c^
F (6,14)	100.129	110.814	46.475	18.418
P	< 0.001*	< 0.001*	< 0.001*	< 0.001*

Small letters in the same column indicate the significant differences between means corresponding to formulations according to a Tukey’s test at *P* < 0.001.

*Highly significant.

D.W: distilled water.

**Table 4:** Mortality percentages of *Rhipicephalus sanguineus* nymphs 3 days post exposure to ivermectin and its different nano-formulations.

Formulation	Concentrations (%)			
	0.5	0.25	0.125	0.0625
IVM spanlastics	93.3 ± 6.7^a^	80.0 ± 5.8^a^	56.7 ± 6.7^a^	40.0 ± 5.8^a^
IVM selenium NPs	70.0 ± 5.8^b^	60.0 ± 5.8^b^	33.3 ± 6.7^b^	10.0 ± 0.0^b^
IVM selenium-chitosan NPs	33.3 ± 3.3^c^	0.0 ± 0.0^c^	0.0 ± 0.0^c^	0.0 ± 0.0^b^
Nano-carrier without IVM	0.0 ± 0.0^d^	0.0 ± 0.0^c^	0.0 ± 0.0^c^	0.0 ± 0.0^b^
Control (D.W)	0.0 ± 0.0^d^	0.0 ± 0.0^c^	0.0 ± 0.0^c^	0.0 ± 0.0^b^
F (4,10)	98.250	114.000	38.000	45.000
P value	< 0.001*	< 0.001*	< 0.001*	< 0.001*

Small letters in the same column indicate the significant differences between means corresponding to formulations according to a Tukey’s test at *P* < 0.001.

*Highly significant.

D.W: distilled water.

**Table 5:** Mortality percentages of *Rhipicephalus sanguineus* unfed adults 3 days post-exposure to two ivermectin nano-formulations.

Formulation	Concentrations (%)			
	0.5	0.25	0.125	0.0625
IVM spanlastics	83.3 ± 3.3^a^	60.0 ± 15.3^a^	50.0 ± 5.8^a^	16.7 ± 6.7^ab^
IVM selenium NPs	53.3 ± 3.3^b^	43.3 ± 12.0^ab^	33.3 ± 12.0^a^	20.0 ± 5.8^a^
Nano-carrier without IVM	0.0 ± 0.0^c^	0.0 ± 0.0^b^	0.0 ± 0.0^b^	0.0 ± 0.0^b^
Control (D.W)	0.0 ± 0.0^c^	0.0 ± 0.0^b^	0.0 ± 0.0^b^	0.0 ± 0.0^b^
F (3,8)	307.167	9.912	14.063	5.857
P	< 0.001*	0.005*	0.001*	0.020**

Small letters in the same column indicate the significant differences between means corresponding to formulations according to a Tukey’s test at *P* < 0.05.

*Highly significant, **Significant.

D.W: distilled water.

### Effect of ivermectin nanoformulations against larvae

All formulations demonstrated acaricidal activity against larvae, with mortality rates ranging from 32.4 to 100% after 24 h of exposure. Complete mortality was observed only with larvae treated with 0.5% IVM spanlastics. All four IVM nanoformulations enhanced the toxicity of IVM compared with free IVM. Ivermectin spanlastics were the most effective against larvae, followed by IVM Se-NPs, IVM Cs-NPs, IVM Se-Cs-NCJ, and then free IVM. Larvae treated with distilled water and nanocarrier without IVM recorded 8.4% and 8.3% mortality, respectively (Table 3).

### Effect of ivermectin nanoformulations against nymphs

The IVM spanlastics exhibited the highest acaricidal activity against nymphs, with mortality rates ranging from 40 to 93.3%, followed by IVM Se-NPs, which recorded mortality rates from 10 to 70%. Furthermore, only 0.5% of the IVM Se-Cs-NCJ showed acaricidal activity on nymphs, with a mortality rate of 33.3%. No mortality was observed in nymphs treated with IVM Cs-NPs and free IVM. Moreover, no mortalities were recorded in nymphs treated with distilled water or nanocarrier without IVM (Table 4).

### Effect of ivermectin nanoformulations against unfed adults

The IVM spanlastics were the most effective against adults, followed by IVM Se NPs. The recorded mortality rates were between 16.7 and 83.3% for IVM spanlastics and 20% and 53.3% for IVM Se NPs. The remaining nanoformulations and free IVM did not show any mortality against adult ticks. Moreover, no mortalities were recorded in unfed treated with distilled water or nanocarrier without IVM (Table 5).

### Comparative toxicity of ivermectin nanoformulations on different tick stages

The LC_50_ values were calculated to estimate the comparative toxicity of IVM nanoformulations against the developmental stages of *R. sanguineus* ticks. In larvae, the free IVM had the lowest larvicidal effect, with LC_50_ value of 0.24%. However, IVM spanlastics exhibited the highest larvicidal effect, with an LC_50_ of 0.05%, followed by IVM Se-NPs (LC_50_: 0.10%), IVM Se-Cs-NCJ (LC_50_: 0.18%), and IVM Cs-NPs (LC_50_: 0.16%). In nymphs and unfed adults, only IVM spanlastics and IVM Se-NPs showed acaricidal effects. They recorded LC_50_ values of 0.09% and 0.23% for nymphs, and 0.16% and 0.38% for adults, respectively. Overall, larvae were more susceptible to the tested nanoformulations, followed by nymphs and unfed adults (Table 6).

**Table 6:** Lethal concentration for 50% of individuals (LC_50_) with their confidence limits for different developmental stages of *Rhipicephalus sanguineus* treated with ivermectin and its nano-formulations.

Tick stage	Formulation	LC_50_ (%)	Confidence limit (%)		Slope ± SE
			Lower	Upper	
Larvae	IVM-spanlastics	0.05	0.03	0.07	2.0 ± 0.4
	IVM selenium NPs	0.10	0.09	0.12	2.1 ± 0.2
	IVM chitosan NPs	0.16	0.12	0.20	1.3 ± 0.2
	IVM selenium-chitosan NPs	0.18	0.16	0.21	2.1 ± 0.2
	Free IVM	0.24	0.16	0.44	0.72 ± 0.2
Nymphs	IVM spanlastics	0.09	0.07	0.11	1.9 ± 0.2
	IVM selenium NPs	0.23	0.19	0.27	2.0 ± 0.2
Unfed adults	IVM spanlastics	0.16	0.14	0.19	2.0 ± 0.2
	IVM selenium NPs	0.38	0.28	0.69	1.0 ± 0.2

### In vivo efficacy of ivermectin spanlastics

**Fig. 3:**
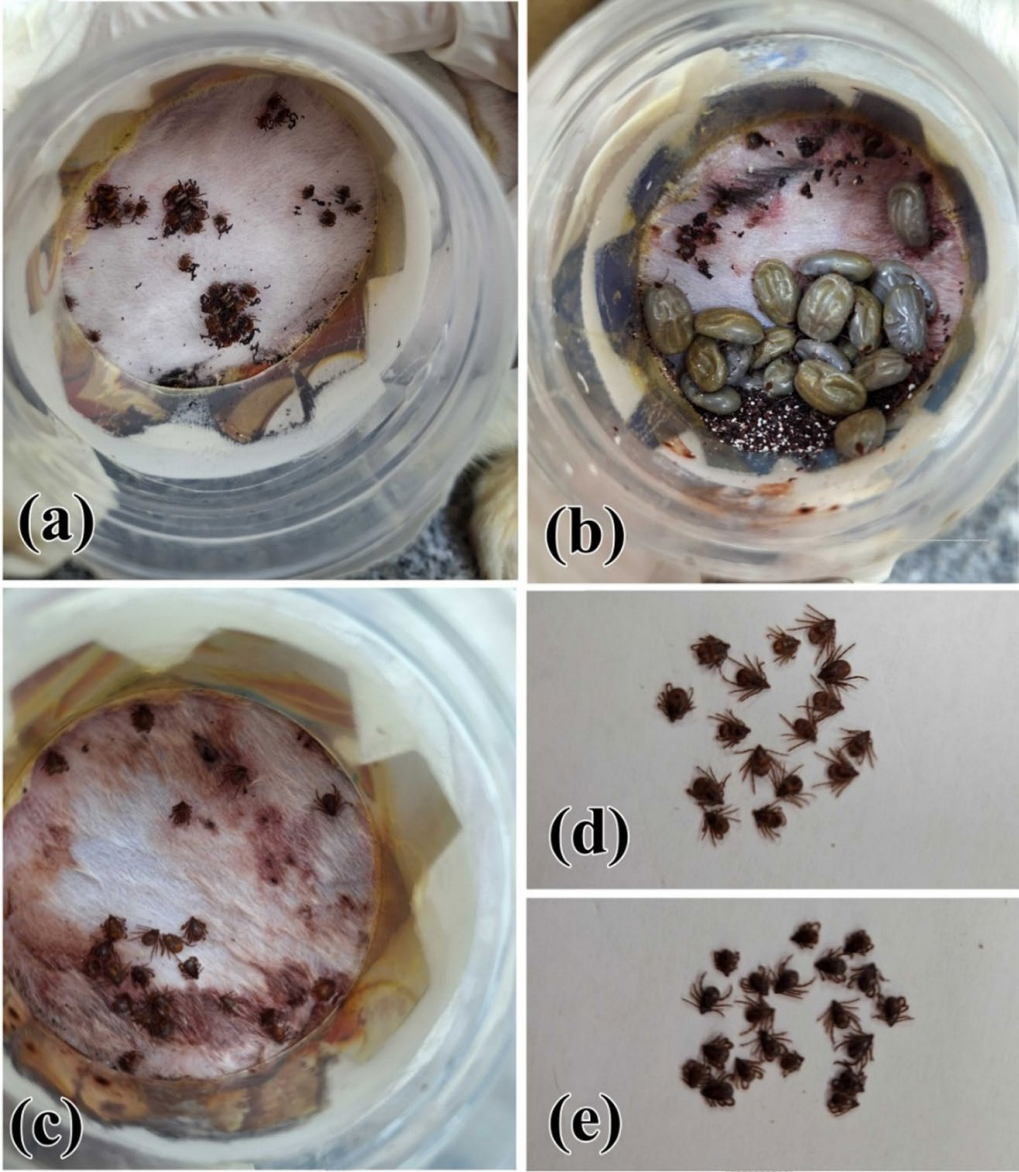
*Rhipicephalus sangeunius* adults fed on rabbits; (**a**,**b**) control group, (**c**–**e**) adult ticks treated with 0.5% of ivermectin spanlastics. (**a**) adult ticks at 0 day of spraying, (**b**) engorged females after completing their feeding, (**c**) dead adults treated with 0.5% of ivermectin spanlastics, (**d**) dead females, (**e**) dead males.

In the current in vivo study, the rabbits experimentally infested with *R. sanguineus* adults treated topically by spraying 0.5% IVM spanlastics exhibited a mortality rate of 2.1%, 87.9%, and 100% on days 1, 3, and 4, respectively, after application compared with the control group. The IVM spanlastics revealed significant acaricidal activity with a decrease in the live ticks within 72–96 h after application. No mortality was recorded in the control group, while all females were fed until full engorgement (Table 7; Fig. 3).

**Table 7:** In vivo efficacy of ivermectin spanlastics (0.5%) on *Rhipicephalus sanguineus* unfed adults fed on rabbits for four days after spraying.

Treatment	Mortality (%)		
	1st day	3rd day	4th day
IVM spanlastics (0.5%)	2.1 ± 0.8#	87.9 ± 2.2*	100 ± 0.0
Control (D.W)	0.0 ± 0.0	0.0 ± 0.0	0.0 ± 0.0
T value	2.500	39.875	
P value	0.130	0.001	

D.W: distilled water, *Highly significant, # non-significant.

## Discussion

The growing resistance to ivermectin, a macrocyclic lactone, coupled with limitations of the conventional injectable formulations in animals, has spurred research into strategies to optimize its antiparasitic potential. For instance, exploring novel delivery systems (like nanocarriers or sustained-release formulations) to be used in the form of an immersion bath or spray provides an additional delivery route alternative to the conventional injection method, enhances efficacy, and improves safety profiles. Additionally, combining ivermectin with other agents or targeting specific parasite life stages may help counter resistance mechanisms and broaden its therapeutic impact^44^. Innovations such as nanoencapsulation, sustained-release implants, and modified-release formulations have shown promise in improving bioavailability, targeting specificity, and reducing dosing frequency, thereby maximizing therapeutic outcomes and minimizing resistance development^45^. Some studies have revealed resistance of ticks to IVM treatment, so higher concentrations have been used to combat this resistance, as in the studies by Sarli et al. and Nava et al.^46,47^ used 3.15% of IVM against *Rhipicephalus microplus* tick, and Zeeshan et al.^48^ used 2.5% IVM against adult *Hyalomma anatolicum* ticks. Therefore, the development of these nanoformulations provides an additional application strategy for IVM. By improving its physicochemical characteristics, the nano-system transforms IVM into an effective topical acaricide, extending its efficacy beyond the conventional injectable method.

The current research presents the fabrication of different nanoformulations loaded with IVM to improve its therapeutic activity. Additionally, the acaricidal activity of these formulations (IVM spanlastics, IVM Se-NPs, IVM Cs-NPs, and IVM Se-CsNPs) was evaluated against larvae, nymphs, and unfed adults of *R. sanguineus* ticks. The prepared nanoformulations were found to be in the nanosized range, which enhances their activity, especially for the smallest particle size IVM spanlastics (450 ± 11.5 nm). Furthermore, the observed lower PDI value (0.34 ± 0.01) for the prepared spanlastics is considered suitable for drug delivery due to the homogeneous distribution of small particles. Also, IVM spanlastics exhibit a significantly higher zeta-potential value (-30 ± 1.9 mV) compared to other formulations, confirming the physical stability of the prepared nanovesicles. The higher value of encapsulation efficiency also indicates that the spanlastics type is the most appropriate nanoplatform for encapsulating a higher amount of IVM. These results may be due to the presence of span 60 in the nanovesicular structure^49^.

Drug-release analysis was conducted to predict the in vivo performance of IVM in nanoplatforms relative to the pure drug. Here, the release profile of IVM from spanlastics showed a sustained, prolonged release over 48 h. This finding is likely due to the hydrophobic nature and high transition temperature of Span 60, which allows for a less permeable nanocarrier structure that leads to a slower rate of drug release. This result aligns with previous studies of Sabra et al. and Halawani et al.^49,50^. The kinetic results showed that Higuchi diffusion was the best-fitted model for IVM release from selected spanlastics. The Korsmeyer-Peppas model was used to determine the release exponent (n), elucidating the drug release mechanism. The n values ranged from 0.367 to 0.6, indicating anomalous (non-Fickian) transport. This suggests IVM release is governed by a combination of diffusion (drug movement through the matrix) and polymer erosion/relaxation, implying both concentration gradient-driven diffusion and structural changes in the nanocarrier contribute to the release profile. These findings align with previous studies of Mohamed et al.^51^.

In this study, all tested IVM nanoformulations exhibited acaricidal activity against larvae, but only IVM spanlastics and IVM Se-NPs showed acaricidal activity against nymphs and unfed adults. Ivermectin spanlastics exhibited the highest acaricidal activity with LC_50_ values of 0.05, 0.09, and 0.16% for larvae, nymphs, and unfed adults, respectively, compared with other IVM nanoformulations. Free IVM showed acaricidal activity against larvae (LC_50_=0.24%) but did not record any mortality against nymphs and unfed adults. Previous research suggests that the greater impact of tested formulations on larvae compared to adult stages may be attributed to adults having a thicker cuticle than larvae, and larvae breathe through their cuticular membranes, which likely allows for greater penetration and absorption of the formulations^52^. The increased susceptibility of larvae compared to adult ticks in our study is consistent with the findings of Castro-Saines et al.^53^, who demonstrated that morphological and histometric damage to the cuticle of *Rhipicephalus microplus* after treatment with *Serratia marcescens* strain EC-35 was more pronounced in the larval stage than in adults, thereby enhancing the efficacy of the treatment. This suggests a consistent pattern across tick species, in which juvenile stages exhibit greater susceptibility to biological and chemical control agents.

The superiority of the IVM spanlastics in this study over other IVM nanoformulations is due to their smaller particle sizes, lower PDI, and higher encapsulation efficiency. Moreover, the physicochemical properties enable controlled drug release and extended contact at the target site, leading to higher mortality rates in *R. sanguineus* ticks^27^. Additionally, the surfactant and elastic matrix of spanlastics increase skin adhesion^27,54^, which facilitates diffusion through the cuticle of the tick and extended delivery of drugs. These features also explain the observed higher mortalities of IVM spanlastics over other IVM formulations.

To the best of our knowledge, no published studies have investigated the use of nano-formulated IVM specifically for ticks, except for one study conducted by Ayoob et al.^55^, who evaluated the effect of IVM loaded on multi-walled carbon nanotubes (MWCNTs) on the *Rhipicephalus annulatus* female tick, where the mortality was 100% compared with free IVM, 23.3% after 72 h at a concentration of 250 µg/mL. IVM nanoformulation with different nanocarriers was studied against parasites other than ticks, as in the study by Ahmadpour et al.^56^ demonstrated that nano-lipid carriers (NLCs) loaded with IVM exhibited significantly enhanced efficacy against *Echinococcus granulosus* protoscoleces, achieving up to 100% mortality, surpassing the effectiveness of free IVM. The niosomes and nano-crystals forms of IVM significantly reduce *Trichinella spiralis* adult and larval counts, with niosomal IVM displaying superior activity by reducing inflammation in both jejunal and muscle homogenates^30^. Importantly, IVM exerts its acaricidal effect by disrupting γ-aminobutyric acid (GABA)-dependent neurotransmission in arthropods, causing paralysis and eventual death due to the inability to attach to the host or prolonged cessation of feeding^57^. Although IVM does not affect the GABA system responsible for stimulating salivary fluid secretion, it may influence other GABA-mediated neural pathways in ticks^58^.

While the IVM spanlastics demonstrated promising results against *R. sanguineus* ticks, the IVM Se-NPs also demonstrated acaricidal activity, with a stronger effect on *R. sanguineus* larvae than on nymphs and adults, as observed in IVM spanlastics. The LC_50_ values were 0.10, 0.23, and 0.38% for larvae, nymphs, and unfed adults, respectively. Selenium nanoparticles have also proven to be effective drug delivery carriers, enhancing bioavailability, controlling release, and producing more stable formulations^59^. Ivermectin Se-NPs have a smaller particle size (600 nm) and a low PDI of 0.4, which indicates their good acaricidal activity against *R. sanguineus* ticks. No previous studies have assessed the effect of IVM Se-NPs on ticks. However, Se-NPs possess intrinsic biological activity and can influence parasite viability by disrupting cuticle integrity or interfering with oxidative/antioxidant pathways^60^. Selenium NPs have shown antiparasitic activity against *Leishmania*^61^, *Giardia*^62^, *Trichinella spiralis*^63^, and external parasites such as mosquitoes^64^. Therefore, IVM combined with Se-based nanocarriers can offer carrier-mediated delivery advantages and potentially exhibit additive or synergistic effects. This dual action is likely the reason for the increased acaricidal activity observed in our IVM Se-NP formulations compared to free IVM.

Concerning IVM Cs-NPs and IVM Se-Cs NCJ, they exhibited an effect only against *R. sanguineus* larvae, with LC_50_ values of 0.16 and 0.18%, respectively. In contrast, no effect was observed against nymphs or unfed adults. The impact of both formulations was higher against larvae compared with free IVM (LC_50_=0.24%). The acaricidal activity of IVM CsNPs and IVM Se-Cs NCJ against larvae may be attributed to the higher sensitivity of larvae than nymphs and unfed adults. In addition, the relatively high particle size and PDI values of these are higher than those of other IVM nanoformulations. No published data has evaluated the acaricidal activity of IVM CsNPs and IVM Se-Cs NCJ against ticks; however, IVMCsNPs were evaluated against *Gasterophilus intestinalis* larvae in donkeys, where the treatment combining ivermectin with chitosan nanoparticles produced synergistic anti-parasitic effects, with observable tegument damage and improved host health markers^65^.

After the promising in vitro outcomes, the in vivo study was conducted to confirm the efficacy of the most effective IVM nanoformulation. The results of the in vivo trial using IVM spanlastics resulted in 100% mortality of *R. sanguineus* adult ticks four days after application. These results proved that the topical application of IVM spanlastics was highly effective against *R. sanguineus* ticks compared to the free IVM. This may result from the improved solubility, stability, and sustained release provided by IVM spanlastics formulation. In addition, nanocarriers increase the surface area of the drug, resulting in improved penetration through the skin and bioavailability, thus maintaining the therapeutic level of IVM at the site of parasite attachment for an extended period^28^.

## Conclusion

This study evaluated the acaricidal activity of different IVM nanoformulations against various developmental stages of *Rhipicephalus sanguineus* ticks in comparison to free IVM. IVM spanlastics demonstrate superior acaricidal activity against all stages of *R. sanguineus*, achieving the lowest LC_50_ values (LC_50_: 0.06–0.16%) compared to other nanoformulations and free IVM. The in vivo results of IVM spanlastics showed 100% mortality of adult ticks four days after application. These findings suggest that IVM spanlastics could be used as a topical alternative to conventional injectable IVM. Further toxicological studies are necessary to ensure the safety of these formulations for environmental and veterinary use.

## Data Availability

Data will be made available on request.
